# Epsilon Aminocaproic Acid’s Safety and Efficacy in Pediatric Surgeries Including Craniosynostosis Repair: A Review of the Literature

**DOI:** 10.7759/cureus.25185

**Published:** 2022-05-21

**Authors:** Alexander Bolufer, Takuma Iwai, Caroline Baughn, Alec C Clark, Greg Olavarria

**Affiliations:** 1 College of Medicine, University of Central Florida College of Medicine, Orlando, USA; 2 Pediatric Surgery, University of Central Florida College of Medicine, Orlando, USA; 3 Pediatric Neurosurgery, Orlando Regional Medical Center, Orlando, USA

**Keywords:** pediatric, transfusion, blood loss, craniofacial surgery, craniosynostosis, eaca, amicar, epsilon aminocaproic acid

## Abstract

Craniosynostosis, the premature fusion of skull sutures in children, requires surgical correction. This procedure routinely requires allogeneic blood transfusions, which are associated with multiple risks of their own. Since 2008, antifibrinolytics tranexamic acid (TXA) and epsilon aminocaproic acid (EACA or Amicar) have been widely used. There is literature comparing the two agents in scoliosis and cardiothoracic surgery, but the literature comparing the two agents in pediatric craniofacial surgery (CF) is limited. Tranexamic acid use is more common in pediatric CF surgery and has been thoroughly studied; however, it costs about three times as much as EACA and has been associated with seizures. This study compiles the literature assessing the safety and efficacy of EACA in reducing blood loss and transfusion volumes in children and explores its potential use in pediatric CF surgery.

Papers from 2000 to 2021 regarding the effectiveness and safety of EACA in Pediatric scoliosis, cardiothoracic, and craniosynostosis surgery were reviewed and compiled. Papers were found via searching PubMed and Cochrane databases with the key terms: Epsilon aminocaproic acid, EACA, Amicar, Tranexamic acid, TXA, craniosynostosis, scoliosis, cardiothoracic, and pediatric. Prospective studies, retrospective studies, and meta-analyses were included.

Twenty-nine papers were identified as pertinent from the literature searched. Four were meta-analyses, 14 were retrospective, and 11 were prospective. Of these papers, seven were of cardiac surgery, 12 were of scoliosis, and nine were of craniosynostosis. During our search, EACA has been shown to consistently reduce blood transfusion volumes compared to control. However, it is not as effective when compared to TXA. EACA has a similar safety profile to TXA but has a reduced risk of seizures. There are not many studies of EACA in craniosynostosis repair, but the existing literature shows promising results for EACA's efficacy and safety, warranting more studies.

## Introduction and background

Craniosynostosis, the premature fusion of skull sutures in children, requires surgical correction to ensure normal craniofacial and neurological development of the patient. Despite the use of cell-saver devices and refined surgical techniques, the invasive nature of this surgery routinely leads to significant blood loss. This requires the administration of allogenic blood or blood products to prevent exsanguination. Indeed, exsanguination is the major complication and cause of death associated with craniosynostosis correction [1,2]. To add to this risk, allogenic blood transfusions are associated with risks of their own (infectious and immunologic) [3-5]. Also, pediatric direct donation programs have not proven to be satisfactory and can be associated with wasted or unused blood products [6]. There is a need to find the ideal agent to minimize bleeding during these surgeries.

In 2008, the Food and Drug Administration removed the blood-sparing agent aprotinin from the market after it was associated with death in adult cardiothoracic surgery [7,8]. Following this, the antifibrinolytics tranexamic acid (TXA) and epsilon aminocaproic acid (Amicar, or EACA) became more widely used as blood sparing agents to lower transfusion volumes. TXA and EACA are lysine analogs that bind to lysine receptors on plasminogen, preventing its conversion to plasmin. Therefore, plasmin is prevented from degrading fibrin, enhancing clot formation [9-12].

Despite the increase in the use of these drugs, there exists no standardized protocol for their use in craniosynostosis correction. There exists a large body of literature on TXA for craniosynostosis repair [13-20]. However, TXA has been associated with seizures [21-23], thrombosis [24,25], and costs about three times as much as EACA [26,27]. Therefore, there has been recent interest in using EACA instead of TXA. However, there exists no current comprehensive review of the literature on the use of EACA for craniosynostosis. The authors are particularly interested in comparing EACA and TXA. The goal of this project is to compile, compare, and analyze the current literature on EACA to evaluate the scientific consensus on EACA’s potential safety and efficacy in craniosynostosis surgery. 

## Review

Methods

The studies included in this review are prospective studies, retrospective studies, and meta-analyses. Searches were conducted in the PubMed and Cochrane databases. The key terms: epsilon aminocaproic acid, EACA, Amicar, tranexamic acid, TXA, craniosynostosis, scoliosis, cardiothoracic and pediatric were used. The references of each paper were also examined, and pertinent ones were included. The review was conducted between August 1, 2021, and September 30, 2021. A single author conducted the search, and each paper was reviewed by at least three authors to ensure each paper fit the inclusion criteria. One author collated each study into a single table. Authors were assigned to cardiac, spinal, and craniosynostosis studies and then collated the data. One other author reviewed each table to ensure accuracy. The consensus was met about conclusions, and then the results were written into the discussion. 

Papers published between the years 2000-2021 were used. Prospective studies, retrospective studies (i.e., chart review), and meta-analyses were included. Papers comparing EACA to TXA were included. Only papers in English were included. Any papers that do not assess the efficacy or safety of EACA at reducing blood loss and transfusion volumes in children or adolescents were excluded. Preliminary studies were excluded.

Results

A total of 29 papers were included for consideration; four were meta-analyses, 14 were retrospective, and 11 were prospective. Of these papers, seven were of cardiac surgery, 12 were of scoliosis, nine were of craniosynostosis, and one did not fit into any other category. Eleven studies compared TXA and EACA. The primary reason a study was excluded was that either the efficacy of EACA was not being evaluated, or it was not studying a pediatric population. All papers included in the study were collated in Table 1. For reference, a detailed summary of EACA in non-craniosynostosis surgeries is compiled in Table 2. Finally, a detailed summary of EACA in craniosynostosis surgeries is compiled in Table 3. 

**Table 1:** All Study Characteristics EACA: Epsilon Aminocaproic Acid, TXA: Tranexamic Acid

Report and Year of Publication	Study Type	Study Size (N)	Type of Intervention(s)	Intervention(s) (N)	Control (N)
Borst et al. [28] 2021	Retrospective	95	EACA vs TXA	47 (EACA), 48 (TXA)	-
Kurlander et al. [29] 2020	Retrospective	39	EACA vs. EACA + EPO	11 (EACA), 9 (EACA + EPO)	19
Nguyen et al. [30] 2019	Retrospective	53	EACA vs. Placebo	23	30
Lam et al. [31] 2019	Retrospective	74	Low vs high dose EACA	36 (low dose), 38 (high dose)	-
Karimi et al. [32] 2019	Meta-Analysis	285	EACA vs TXA vs Control	EACA (61), TXA (101)	123
Lonner et al. [33] 2018	Retrospective	1,769	EACA vs. TXA vs. Control	117 (EACA), 525 (TXA)	1,127
Thompson et al. [34] 2017	Retrospective	43	EACA vs. Control	14	29
Goobie et al. [35] 2017	Retrospective	1,638	EACA vs. TXA vs. Control	383 (EACA), 591 (TXA)	664
Reddy et al. [36] 2016	Prospective	2	EACA only	2	-
Hsu et al. [37] 2016	Retrospective	152	EACA vs. Control	66	86
Lu et al. [38] 2015	Meta-Analysis	515	EACA vs. Placebo	248	267
Stricker et al. [39] 2015	Prospective	18	EACA only	18	-
Wang et al. [40] 2015	Meta-Analysis	1,158	Antifibrinolytics (EACA, Aprotinin, TXA) vs. Placebo	613	545
McLeod et al. [41] 2015	Retrospective	4,269	EACA vs TXA	155 (EACA), 72 (TXA)	-
Verma et al. [42] 2014	Prospective	125	EACA vs. TXA vs. Placebo	42 (EACA), 36 (TXA)	47
Oppenheimer et al. [43] 2014	Retrospective	148	EACA vs. Control	30	118
Halanski et al. [44] 2014	Prospective	47	EACA vs. TXA	25	22
Scott et al. [45] 2014	Retrospective	145	EACA vs Aprotinin	68 (Aprotinin), 77 (EACA)	-
Stricker et al. [46] 2013	Prospective	18	EACA	18	-
Pasquali et al. [47] 2012	Meta-Analysis	22,258	EACA vs. TXA vs. Aprotinin vs. Placebo	1,667 (EACA), 7,329 (Aprotinin), 1,486 (TXA)	11,766
Dhawale et al. [48] 2012	Retrospective	84	EACA vs. TXA vs. Placebo	14 (EACA), 30 (TXA)	40
Martin et al. [49] 2011	Prospective	105	EACA vs. TXA	77	28 (TXA)
Martin et al. [50] 2011	Prospective	234	EACA vs. TXA	120	114 (TXA)
Thompson et al. [51] 2008	Retrospective	73	EACA vs. TXA	57	16 (TXA)
Thompson et al. [52] 2008	Retrospective	96	EACA vs. Control	62	34
Thompson et al. [53] 2007	Prospective	51	EACA	51	-
Florentino-Pined et al. [54] 2004	Prospective	59	EACA vs. Control	28	31
Chauhan et al. [55] 2004	Prospective	150	EACA vs. TXA vs. Control	50 (EACA), 50 (TXA)	50
Rao et al. [56] 2000	Prospective	170	EACA vs. Control	85	85

**Table 2:** Amicar (EACA) and/or TXA Use in Non-Craniosynostosis Surgeries EACA: Epsilon Aminocaproic Acid, TXA: Tranexamic Acid, MAP: Mean Arterial Pressure, PTT: Partial Thromboplastin Time, INR: International Normalized Ratio

Indication	Reference (Study Type)	Intervention(s) Analyzed (Sample Size)	Primary Outcome	Other Notes
Cardiac Surgery (N=7)	Lu et al. [38] 2015 (Meta-analysis)	EACA vs. Placebo (N=515)	Postoperative blood loss mean difference compared to placebo: -7.08 mL; 95% CI: -16.11 to 1.95; P=0.12	Two fatal cases of thrombosis. Analyzed trials used different dosing regimens.
	Scott et al. [45] 2014 (Retrospective)	EACA vs. Aprotinin (N=145)	Infants on EACA required significantly more rFVIIa P<0.001	Bleeding in infant cardiac surgery increased in the switched from aprotinin to EACA.
	Pasquali et al. [47] 2012 (Retrospective)	TXA vs. EACA vs. Aprotinin (N=22,258)	Overall, in-hospital mortality rate TXA: 2%; EACA: 3.9%; Aprotinin: 4% P=0.009	TXA provided the best outcomes. Dosing regimens used were not fully provided. No difference in blood loss in EACA vs. aprotinin.
	Martin et al. [50] 2011 (Prospective)	TXA vs. EACA (N=234)	24-hour postoperative blood loss TXA: 21 mL/kg (14 - 38) EACA: 29 mL/kg (14 - 40) P=0.242	Dosing was standardized in this study. Seizure rate was significantly lower is EACA group. Renal dysfunction, but not renal failure or in-hospital mortality, was significantly lower in TXA group.
	Martin et al. [49] 2011 (Prospective)	TXA vs. EACA (N=105)	24-hour postoperative blood loss TXA: 39 mL/kg; EACA: 41 mL/kg P=0.625	This study was conducted exclusively on neonates. Dosing was standardized in this study.
	Chauhan et al. [55] 2004 (Prospective)	TXA vs. EACA vs. Control (N=150)	24-hour postoperative blood loss EACA: 28 ± 13 mL/kg; TXA: 27 ± 14 mL/kg P>0.05	No complications in the form of renal failure or neurologic events were reported.
	Rao et al. [56] 2000 (Prospective)	EACA vs. Control (N=170)	24-hour postoperative blood loss EACA: 23.7 +/- 5.8 mL/kg Control: 42.6 +/- 6.9 mL/kg P<0.001	EACA significantly reduced packed red cells and platelet concentrate.
Spinal Surgery (N=12)	Lam et al. [31] 2019 (Retrospective)	Low (10mg/kg/hr) vs. High Dose (33 mg/kg/h) EACA (N=74)	High dose EACA was associated with a greater intraoperative blood loss of 8.1 mL/kg P=0.009	Authors noted that EACA does not appear to have a dose dependent effect, which is backed by prior studies.
	Lonner et al. [33] 2018 (Prospective)	TXA vs. EACA vs. Control (N=1,769)	Estimated Blood Loss TXA: 742.3 mL; EACA: 1,420.6 mL; Control 1,010.6 mL P<0.0001	This was a multicenter, multi-surgeon study.
	McLeod et al. [41] 2015 (Retrospective)	EACA vs. TXA vs. Aprotinin (N= 4,269)	EACA reduced odds of transfusion in adolescent idiopathic scoliosis. (OR=0.4, P<0.001). No reduction in transfusion with TXA	Neither TXA nor EACA reduced odds of transfusion in neuromuscular scoliosis. Number of vertebrae fused correlates to odds of needing transfusion. Overall use of antifibrinolytics still unclear.
	Stricker et al. [39] 2015 (Prospective)	Various regimens of EACA (N= 20)	Optimal regimen based on weight (loading dose and infusion rate) <25 kg: 100 mg/kg; 40 mg/kg/hr 25-50 kg: 100 mg/kg; 35 mg/kg/hr ≥ 50 kg: 100 mg/kg; 30 mg/kg/hr	Weight, age, and perioperative conditions can influence EACA pharmacokinetics. The authors recommended employing an efficacy trial to evaluate this dosing regimen.
	Wang et al. [40] 2015 (Meta-analysis)	TXA vs. EACA vs. Apronitin vs. Placebo (N=1,158)	Mean difference of total blood loss compared to placebo TXA: −828.60 mL P=0.0004 EACA: −329.34 mL P=0.004	One pulmonary embolism was reported in TXA group. No adverse events were reported in the EACA group.
	Halanski et al. [44] 2014 (Prospective)	TXA vs. EACA (N=47)	Mean volumes transfused TXA: 461 mL; EACA: 1,014 mL; P=0.03	TXA group demonstrated a statistically significant smaller change in INR, a lower PTT, and greater fibrinogen levels postoperatively.
	Verma et al. [42] 2014 (Prospective)	TXA vs. EACA vs. Placebo (N=125)	Mean blood loss TXA: 783 ± 514 mL; EACA: 493 ± 120 mL; Control: 960 ± 175 mL	Maintaining MAP at <75 mm Hg contributed to less blood loss. TXA and EACA blood loss similar, neither affected transfusion rate.
	Dhawale et al. [48] 2012 (Retrospective)	TXA vs. EACA vs. Placebo (N=84)	Mean blood loss TXA: 1,301 mL; EACA: 2,502 mL; Control 2,684 mL P<0.001	No adverse events were reported.
	Thompson et al. [51] 2008 (Retrospective)	EACA vs. Control (N=73)	Total perioperative blood loss EACA: 2,095.7 ± 952.3 mL; Control: 3,442.8 ± 1,344 mL P=0.001	No complications were reported due to EACA use.
	Thompson et al. [52] 2008 (Retrospective)	EACA vs. Control (N=96)	Intraoperative blood loss EACA: 1,125 ± 715 mL; Control: 2,194 ±1,626 mL P<0.0002	No complications were reported due to EACA use.
	Thompson et al. [53] 2007 (Prospective)	Fibrinogen levels following EACA administration (N=51)	Preoperative fibrinogen levels were 255.5 mg/dL, rose through postoperative period to 680.9 mg/dL on fifth day	Authors noted significance is unknown.
	Florentino et al. [54] 2004 (Prospective)	EACA vs. Control (N=36)	Total perioperative blood loss EACA: 1,391 ± 212 mL; Control: 1,716 ± 513 mL P=0.036	No complications were reported due to EACA use.
Other	Karimi et al. [32] 2019 (Meta-Analysis)	EACA vs. TXA vs Control (N=285)	Antifibrinolytics are effective at reducing perioperative and intraoperative blood loss and transfusion volumes	While EACA does reduce blood loss it was not statistically significant. Authors unsure if due to less potency or insufficient study.

**Table 3:** Amicar Use in Craniosynostosis Surgeries (N=9) EACA: Epsilon Aminocaproic Acid, TXA: Tranexamic Acid, CIVI: Continuous Intravenous Infusion, EPO: Erythropoietin

Reference (Study)	Sample Size	Intervention(s) Analyzed	Primary Outcome	Other Notes
Borst et al. [28] 2021 (Retrospective)	N=95	EACA vs TXA	Calculated Blood Loss (ml/kg) 35±24 (EACA) and 33±18 (TXA) P=0.827	No difference in intraoperative or perioperative blood loss or complications. Authors recommend using the fibrinolytic most cost effective, which in most cases is EACA
Kurlander et al. [29] 2020 (Retrospective)	N=39	EACA vs. EACA + Erythropoietin (EPO) vs. Control	Estimated blood loss EACA: 16.7 mL/kg Control: 47.9 mL/kg P<0.05	Transfusion free discharge rate: EACA: 27%; EACA + EPO: 66% Estimated Blood loss EACA: 16.7 mL/kg EACA + EPO: 11.7 mL/kg P = 0.82
Nguyen et al. [30] 2019 (Retrospective)	N=53	High infusion rate (40 mg/kg/h) vs. a low infusion rate (≤30 mg/kg/h) EACA	Median decrease in intraoperative blood loss for high infusion EACA: 14.32 mL/kg (95% CI 6.64-23.92), P <0.001	This corresponds to about 15% of the child’s total blood volume.
Thompson et al. [34] 2017 (Retrospective)	N=43	EACA vs. Control	Patients receiving EACA had reduced blood loss (P=0.005) and reduced blood transfusion requirement (P=0.010).	These results apply to shorter surgical cases.
Goobie et al. [35] 2017 (Meta-analysis)	N=1,638	EACA vs. TXA vs. Control	Post-operative incidence of seizures or seizure-like events. TXA: 0.34%; EACA: 1.04%; Control: 0.60%	One reported case of Deep Vein Thrombosis in TXA patient
Reddy et al. [36] 2016 (Prospective)	N=2	EACA + EPO	Estimated blood loss from two cases: 43 mL/kg and 19 mL/kg Institution average estimated blood loss for non-EACA cases: 63 mL/kg	EACA may be indicated in certain religious scenarios where patients deny transfusions.
Hsu et al. [37] 2016 (Retrospective)	N=152	EACA vs. Control	Calculated blood loss EACA: 82 ± 43 mL/kg Control: 106 ± 63 mL/kg P=0.01	Post-operative 24 h surgical drain output EACA: 28 mL/kg Control: 37 mL/kg P = 0.001
Oppenheimer et al. [43] 2014 (Retrospective)	N=148	EACA vs. Control	Perioperative transfusion volume EACA: 25.5 mL/kg Control: 53.3 mL/kg P<0.0001	Percentage of patients requiring a second unit of blood EACA: 21% Control: 43%, P < 0.0001 Intraoperative estimated blood loss EACA:322 mL Control: 327 mL P > 0.05
Stricker et al. [46] 2013 (Prospective)	N=6	3 different EACA dose regimens	Optimal regimen for 6 – 24-month-olds: loading dose of 100 mg/kg followed by a CIVI of 40 mg/kg/h	Weight, age, and perioperative conditions can influence EACA pharmacokinetics

Discussion

EACA in Pediatric Cardiac Surgery

In the two prospective studies comparing EACA and TXA from 2011 by Martin et al. [49,50], EACA had slightly higher rates of blood loss than TXA, although this was not statistically significant (P=0.242, P=0.625). No significant difference in perioperative or intraoperative complications was appreciated. The other prospective study comparing TXA and EACA, performed in 2004 by Chauhan et al., also reported no significant difference in blood loss [55]. The final prospective study, performed in 2000 by Rao et al., compared EACA vs. control and appreciated a dramatic and statistically significant reduction in blood loss (EACA: 23.7 +/- 5.8 mL/kg, Control: 42.6 +/- 6.9 mL/kg, P<0.001) [56].

A 2014 single-center retrospective study by Scott et al. compared their institution’s experiences with EACA versus aprotinin after they ceased their aprotinin use in 2007. They found EACA to be associated with increased median volumes transfused for certain blood transfusion products in infant surgery with cardiopulmonary bypass (CPB). The authors state this is because EACA lacks certain platelet stabilizing effects. Those being intraoperative platelets (0 vs. 28, P=0.005, adjusted P=0.06), perioperative Packed Red Blood Cells (40 vs. 60, P=0.02, adjusted P=0.22), fresh frozen plasma (22 vs. 40, P=0.07, adjusted P=0.60) and total volume (73 vs. 135, P=0.04, adjusted P=0.28). They found no increase in events of mortality, renal failure, CNS events, or thrombosis. However, EACA was associated with an increased incidence of post-CPB surgical re-exploration (7 vs. 21, P=0.01), rFVIIa infusion (3 vs. 19, P<0.001), and Inhaled Nitric Oxide (iNO) use (1 vs. 8, P=0.04). The authors explain that their antifibrinolytic use was not randomized, their subgroup size was small, and this was just a single institutional experience. These limiting factors may explain the higher rates of post-surgical exploration and complications in the EACA group [45].

In another retrospective study by Pasquali et al. consisting of 25 centers and 22,258 patients between 2004 and 2008, EACA and aprotinin were similar in overall efficacy and safety, except EACA was associated with greater mortality in patients who needed re-operation, 2.59 (1.04-6.45, P=0.04), and with neonates who had bleeding requiring surgical intervention, 2.81(1.12-7.09, P=0.03). Compared to aprotinin, TXA’s overall in-hospital mortality was found to be significantly better at 0.39 (0.21-0.71, P=0.002). The authors noted that the higher complication and mortality profile of EACA may have been due to the limitations of their study, such as small populations in patient subgroups, institutional variation in the protocol, and missing data such as dosing or indications for medication [47].

Finally, a 2015 meta-analysis by Lu et al. analyzed five prospective studies from Asia (Total N=515), which compared EACA versus placebo. EACA reduced mean transfusion volumes by a mean of -7.08 mL (95% CI: -16.11 to 1.95; P=0.12); however, two instances of fatal thrombosis were reported. Authors recommend that EACA be used, but only in instances where it is essential [38].

EACA in Pediatric Spinal Surgery

Studies comparing TXA and EACA found TXA to be superior at reducing estimated blood loss and transfusion volumes [33,40,44,48], except for McLeod et al. [41] and Verma et al. [42]. In a retrospective study of 37 institutions conducted by McLeod et al., EACA was found to be associated with a lower incidence of transfusion (OR=0.4, P<0.001) in Adolescent Idiopathic Scoliosis. There was no difference between TXA and EACA in Neuromuscular scoliosis. However, the strongest association with transfusion volumes was the number of vertebrae fused. Verma et al. found no difference between TXA and EACA in estimated blood loss; however, total postoperative drainage volumes were lower with TXA, and when compared to saline, post-operative Hematocrit was reduced with EACA but not with TXA (P<0.001 and P= 0.011, respectively) [40].

Studies of EACA versus control found EACA to statistically reduce intraoperative blood loss without complications [51,52,54]. Lam et al. conducted a retrospective analysis of 74 patients at a single institution to see if larger doses of EACA are associated with less blood loss [31]. Interestingly, their data suggests the contrary. High dose EACA (33 mg/kg/hr) was associated with a greater intraoperative blood loss of 8.1 mL/kg than the low dose (10 mg/kg/hr, P=0.009). The authors believe this result may be because EACA interferes with platelet-vessel interactions. Finally, in 2007 Thomson et al. performed a prospective study measuring post-operative fibrinogen levels in patients who received EACA. Preoperative fibrinogen levels were 255.5 mg/dL and rose through the postoperative period to 680.9 mg/dL on the fifth day. The others noted that they were unsure of the significance of this finding [53].

EACA in Craniosynostosis Surgery

A recent 2021 retrospective study by Borst et al., N=95, compared TXA with EACA and concluded that there is no difference in efficacy or complications [28]. With these findings, Borst et al. recommended using the more cost-effective anti-fibrinolytic, which is EACA in most instances. Likewise, meta-analyses of 31 institutions (N=1,638) by Goobie et al. found no statistically significant difference in the incidence of seizures attributable to TXA, EACA, or control use. However, it is worth noting that one patient who received TXA was found to have suffered a Deep Vein Thrombosis [35].

Additionally, studies of EACA versus control have demonstrated EACA to be efficacious and safe, with a statistically significant reduction in blood loss or perioperative transfusion volume noted in each study [29,34,37,43]. The dosing effects of Amicar in craniosynostosis have also been analyzed in a retrospective study by Nguyen et al. [30] and a prospective study by Stricker et al. [46]. These studies found EACA to be most efficacious if the patient is given a 100 mL loading dose followed by a relatively high continuous intravenous infusion (CIVI) of 40 mg/kg/hr, as opposed to lower dosing regiments. This finding is contrary to the experience of Lam et al., who did not find the efficacy of EACA to be dose-dependent in spinal surgeries [31]. More studies will need to be conducted to determine if this dose-dependent effect is only a phenomenon observed in spinal surgeries and not craniosynostosis surgeries.

Finally, Kurlander et al. [29] and Reddy et al. [36] both documented the effect of perioperative administration of erythropoietin along with EACA use. Although Kurlander et al. noted no statistically significant difference in estimated blood loss when comparing groups given EACA and erythropoietin versus EACA alone (P=0.82), they did note an increase in the transfusion free rate (66% in the EACA+ EPO group versus 27% in the EACA group). Reddy et al. documented two uncommon cases involving patients who declined blood transfusions due to their religious beliefs. In both of these cases, co-administration of EACA and erythropoietin led to both patients experiencing less blood loss than the institution average. These findings suggest that concurrent administration of erythropoietin may provide additional benefits when administered with EACA. More research will need to be conducted to explore and potentially optimize this protocol.

## Conclusions

EACA has been used for a variety of indications ranging from cardiothoracic surgery to spinal surgery and, more recently, for craniosynostosis surgery. Although EACA showed effectiveness at reducing blood loss when compared to controls, it did not consistently reduce blood loss when compared to TXA. It is worth noting that the protocols implemented for EACA, such as the loading dose and infusion rates, were often not standardized, which may have contributed to the unfavorable data obtained. On the other hand, the data on EACA use in craniosynostosis surgery suggests that EACA is associated with a lower risk of seizures and provides comparable outcomes with a similar safety profile as TXA. As the literature involving EACA use in craniosynostosis is limited, further studies investigating its optimal dosing, interactions with erythropoietin, as well as other effects are necessary to elucidate the true applications of this therapeutic agent.
